# ACCEPT 2·0: Recalibrating and externally validating the Acute COPD exacerbation prediction tool (ACCEPT)

**DOI:** 10.1016/j.eclinm.2022.101574

**Published:** 2022-07-22

**Authors:** Abdollah Safari, Amin Adibi, Don D. Sin, Tae Yoon Lee, Joseph Khoa Ho, Mohsen Sadatsafavi

**Affiliations:** a Respiratory Evaluation Sciences Program, Collaboration for Outcomes Research and Evaluation, Faculty of Pharmaceutical Sciences, University of British Columbia, Vancouver, Canada; b Department of Mathematics, Statistics, and Computer Science, University of Tehran, Tehran, Iran; c Centre for Heart Lung Innovation, St. Paul's Hospital and Department of Medicine (Division of Respirology), The University of British Columbia, Vancouver, Canada

**Keywords:** Clinical prediction models, Chronic obstructive pulmonary disease, Prognosis, Precision medicine

## Abstract

**Background:**

The Acute Chronic Obstructive Pulmonary Disease (COPD) Exacerbation Prediction Tool (ACCEPT) was developed for individualised prediction of COPD exacerbations. ACCEPT was well calibrated overall and had a high discriminatory power, but overestimated risk among individuals without recent exacerbations. The objectives of this study were to 1) fine-tune ACCEPT to make better predictions for individuals with a negative exacerbation history, 2) develop more parsimonious models, and 3) externally validate the models in a new dataset.

**Methods:**

We recalibrated ACCEPT using data from the Evaluation of COPD Longitudinally to Identify Predictive Surrogate End-points (ECLIPSE, a three-year observational study, 1,803 patients, 2,117 exacerbations) study by applying non-parametric regression splines to the predicted rates. We developed three reduced versions of ACCEPT by removing symptom score and/or baseline medications as predictors. We examined the discrimination, calibration, and net benefit of ACCEPT 2·0 in the placebo arm of the Towards a Revolution in COPD Health (TORCH, a three-year randomised clinical trial of inhaled therapies in COPD, 1,091 patients, 1,064 exacerbations) study. The primary outcome for prediction was the occurrence of ≥2 moderate or ≥1 severe exacerbation in the next 12 months; the secondary outcomes were prediction of the occurrence of any moderate/severe exacerbation or any severe exacerbation.

**Findings:**

ACCEPT 2·0 had an area-under-the-curve (AUC) of 0·76 for predicting the primary outcome. Exacerbation history alone (current standard of care) had an AUC of 0·68. The model was well calibrated in patients with positive or negative exacerbation histories. Changes in AUC in reduced versions were minimal for the primary outcome as well as for predicting the occurrence of any moderate/severe exacerbations (ΔAUC<0·011), but more substantial for predicting the occurrence of any severe exacerbations (ΔAUC<0·020). All versions of ACCEPT 2·0 provided positive net benefit over the use of exacerbation history alone for some range of thresholds.

**Interpretation:**

ACCEPT 2·0 showed good calibration regardless of exacerbation history, and predicts exacerbation risk better than current standard of care for a range of thresholds. Future studies need to investigate the utility of exacerbation prediction in various subgroups of patients.

**Funding:**

This study was funded by a team grant from the Canadian Institutes of Health Research (PHT 178432).

**Research in context**

**Evidence before this study**

Lack of external validation is a widespread phenomenon for clinical prediction models, especially those in Chronic Obstructive Pulmonary Disease (COPD). Recent systematic reviews have shown that COPD prediction models are validated on average 0·09 times per model in comparison to 1·30 per model for cardiovascular disease models. The Acute COPD Exacerbation Prediction Tool (ACCEPT) is a recently developed risk prediction model for the risk and severity of COPD exacerbations; however, its development sample included only patients with a positive exacerbation history. Consequently, while ACCEPT was well calibrated in these patients, it overestimated the risk in individuals with no exacerbations in the preceding year.

**Added value of this study**

In this study, we recalibrated ACCEPT to correct for overestimation of risk in non-exacerbators and developed more parsimonious versions of the model to facilitate ease of clinical use. The resulting full and reduced versions of ACCEPT 2·0 were externally validated in a new dataset, including evaluating the net benefit of the updated models compared with exacerbation history alone. ACCEPT 2·0 shows promise in conferring higher clinical utility than the current standard of care.

**Implications of all the available evidence**

Continuous updating and validating existing clinical prediction models are crucial for ensuring they provide clinical utility. The next step is to investigate the validity of this tool in different COPD populations (e.g., by country) and conduct real-world impact studies that directly compare the outcomes of risk scoring tools like ACCEPT against current standard of care.

Alt-text: Unlabelled box

## Introduction

An important component of the contemporary clinical management of chronic obstructive pulmonary disease (COPD) is the prevention of flare-up of symptoms, known as exacerbations or lung attacks.^1^ Current guidelines recommend a step-wise approach for preventive pharmacotherapy based on exacerbation history in the previous 12 months.^2^ This is based on the widely accepted notion that the best predictor of future exacerbation is the previous history of exacerbations.^3^ However, other patient and disease characteristics can also predict exacerbation risk, thus adding to the accuracy of predictions. Indeed, a recent study has shown that relying on exacerbation history alone for stepping up or down treatments is not much different than changing treatments at random.^4^

We recently published the Acute COPD Exacerbation Prediction Tool (ACCEPT), a clinical prediction model that predicts individualised rate, risk, and severity of moderate/severe exacerbations based on clinical and demographic variables.^5^ However, the development sample for ACCEPT was three randomised clinical trials (RCTs), all of which required patients to have a positive exacerbation history as an inclusion criterion. Consequently, in external validation, while ACCEPT was well calibrated in general, it overestimated the risk in individuals with no exacerbations in the preceding year. Further, some of the predictors in ACCEPT, such as the St. George Respiratory Questionnaire (SGRQ) score, are not routinely collected in clinical practice. Parsimonious models with fewer or more easily obtainable predictors would be more desirable for clinical adoption. Finally, in external validation, ACCEPT showed an improvement in discriminatory performance over exacerbation history alone (increase in area under the curve [AUC] of 0·02 for moderate/severe exacerbations and 0·11 for severe exacerbations); however, it is not obvious to what extent these improvements translate to higher clinical utility.^6^

The purpose of this study was to recalibrate ACCEPT to correct for overestimation of risk in non-exacerbators, to develop more parsimonious versions to facilitate clinical use, and to externally validate the resulting ACCEPT 2·0 (full and reduced versions) in a new dataset, including evaluating the net benefit of ACCEPT 2·0 compared with exacerbation history alone (current standard of care).

## Methods

This article is prepared in accordance with the Transparent Reporting of a Multivariable Prediction Model for Individual Prognosis or Diagnosis (TRIPOD) Statement.^7^

### Sources of development, recalibration, and validation data

**Figure 1:**
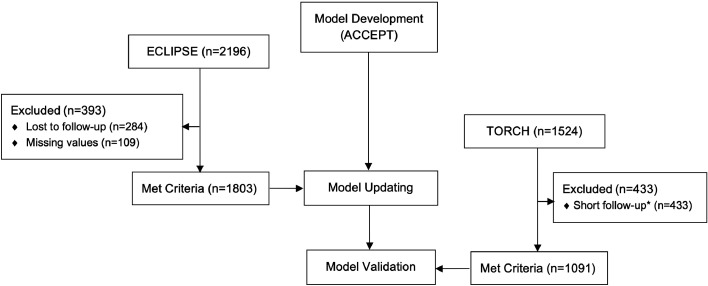
Study flowchart. *short follow-up was defined as <0·3 year; ECLIPSE, Evaluation of COPD Longitudinal to Identify Predictive Surrogate End-points;^11^ TORCH, Towards a Revolution in COPD Health;^12^ ACCEPT, Acute COPD Exacerbation Prediction Tool; COPD, chronic obstructive pulmonary disease.

Figure 1 provides the flow diagram of the study. The datasets used for the development and validation are explained in Section 1 of the Supplementary Material. The development and external validation of ACCEPT 1·0 are explained in detail in its original publication.^5^ In brief, the three datasets used for the development of ACCEPT were from one-year RCTs (MACRO,^8^ STATCOPE,^9^ and OPTIMAL^10^), with a combined sample size of 2,249 (contributing 2,896 events). ACCEPT was externally validated in data from the Evaluation of COPD Longitudinally to Identify Predictive Surrogate End-points (ECLIPSE) study^11^ (*n* = 1,803, with 2,117 events). We excluded patients with short follow-up time (<0.3 years). To assess the potential non-random censoring, we compared the distribution of predicted risks between this subgroup and the rest of the sample.

For the present study, we used data from ECLIPSE for recalibration of ACCEPT. We then validated the resulting ACCEPT 2·0 using the data from the placebo arm of the Towards a Revolution in COPD Health (TORCH, we also considered the treatment arms in a sensitivity analysis).^12^ Unlike the original development sample, ECLIPSE and TORCH had three years of follow-up, which enabled us to use the first-year data (during which patient could have any number of events) for verifying predictor values, and the second-year data for ascertaining the outcome. This in turn enabled us to recalibrate the model in ECLIPSE and evaluate its performance in TORCH that included individuals with negative and positive exacerbation histories.

### Outcomes

The outcome was the 12-month rate or risk of moderate/severe exacerbations. We focused on moderate/severe exacerbations as they are the determinants of disease management in many guidelines, such as the Global Initiative for Chronic Obstructive Lung Disease (GOLD), mild exacerbations for which the patient does not seek medical care are not determinants of COPD management.^2^ All five data sources used event-based exacerbation definitions aligned with the definition adopted by GOLD.^2^ According to this definition, moderate exacerbations are those that require the initiation of systemic corticosteroids with/without antibiotics, and severe exacerbations are those that require an emergency department visit or hospitalisation.

A distinct feature of ACCEPT, compared with previous prediction models,^13^ is that it predicts both the rate and the severity of exacerbations, as well as the risk of any combinations of the two. This is done by the use of a joint frailty-logistic model, with the frailty component modelling the rate of all events, and the logistic component modeling the probability that a given event would be severe (for more details on the model specification, see Section 2 of Supplementary Material).^14^ This in turn enables the quantification of the probability of any given combination of moderate and severe exacerbations. One such pattern is the occurrence of ≥2 moderate or ≥1 severe exacerbations, which is a crucial determinant of pharmacotherapy in the influential GOLD strategy^2^ and in the Canadian guidelines.^15^ Concordantly, the primary outcome was the occurrence of this pattern during the next 12 months. The secondary outcomes were the occurrence of any (≥1) moderate/severe or any (≥1) severe exacerbations.

### Predictors

The default set of predictors in the full ACCEPT 2·0 are based on the original model.^5^ It includes the number of moderate and severe exacerbations in the previous 12 months, age, sex, smoking status, observed versus predicted forced expiratory volume in one second,^16^ SGRQ score, body mass index (BMI), the use of domiciliary oxygen therapy, use of statins (representing cardiovascular disease risk), and type of inhaled COPD medications (as a surrogate for the severity of COPD). The latter medications included long-acting muscarinic receptor antagonists (LAMAs), long-acting β2 agonists (LABAs), and inhaled corticosteroids (ICS) as separate predictors. In addition to this full model, we created ‘reduced’ versions of the model by removing baseline COPD medications, SGRQ score, or both. This was done because SGRQ (which has 50 items) may be difficult to administer at a busy clinic (and that symptom scores are not generally available in Electronic Health Records [EHR]) and initiating pharmacotherapy based on ACCEPT results in a data shift and feedback, affecting the predictive value of medication use in subsequent visits.^17^ These combinations resulted in one ‘main’ and three ‘reduced’ ACCEPT 2·0 models.

### Statistical analysis

#### Model recalibration

To recalibrate ACCEPT using ECLIPSE data, we used a multivariable adaptive regression spline (MARS) model, a non-parametric regression technique.^18^ A MARS model is a connected set of simple regression functions in different region of the predicted risks (which in turn enables capturing potential non-linearities). We used the first year of data in ECLIPSE to verify predictors (including exacerbation history) and the second year for outcome assessment. We passed the predictions from ACCEPT to a spline model where the outcome was the observed exacerbation rates in year two of ECLIPSE. The spline model smoothly adjusted the predictions based on the observed exacerbation patterns, potentially correcting under- or over-estimation. We repeated this approach separately for all (moderate/severe) and severe exacerbations. To avoid overfitting, we used natural spline models with only three knots and with a cubic regression between knots (see Section 3 of Supplementary Material for more details). We did not use more sophisticated methods such as cross-validation for fine-tuning the parameters of the MARS model due to the risk of overfitting.

#### External validation

We examined the performance of ACCEPT 2·0 in terms of calibration, discrimination, and net benefit in TORCH. TORCH was a three-year RCT which evaluated the effect of salmeterol and fluticasone on the rate of moderate/severe exacerbations and mortality in patients with COPD (for more details on the TORCH study, see Table SM1 of Supplementary Material). We used the first-year data to measure predictors, including exacerbation history, and the second-year data to validate the model against observed exacerbation patterns. The placebo arm of TORCH was used in the main analysis, but we performed a similar analysis in the entire data of TORCH in a sensitivity analysis.

No participant received LAMA in TORCH (which was not a permitted concomitant medication for this study). However, setting the value of LAMA in this dataset to zero is inappropriate, as unavailability of a medication for participants is not tantamount to not using the medication if it were available. Therefore, we considered LAMA use as missing and imputed its values based on other predictors using multiple imputation techniques.^19^^,^^20^ Specifically, we used a logistic regression model to estimate the probability of LAMA use conditional on all other predictors based on the pooled data from the four other development/calibration datasets. We then generated a binary value for LAMA use based on the patient's estimated probability. To account for the uncertainty around the imputed values, we performed multiple imputations using ten repetitions. In a sensitivity analysis, we also set the value of LAMA to 0 (no use) for all the patients in TORCH and repeated the analysis. In addition to LAMA, 264 patients had missing SGRQ scores in TORCH. Given the difficulties in collecting SGRQ from patients, it is not unexpected to see more missing values in this score compared to other predictors. Similarly, we used the same imputation approach, this time based on the observed SGRQ scores in other patients in TORCH. The final model predictions were based on the average of predicted values from each of the imputed datasets.

#### Model calibration

To evaluate the extent that the predicted risks are aligned with the actual risks, we drew calibration plots. We grouped individuals into ten subgroups based on the deciles of the predicted rate and calculated the observed rate within each decile based on the observed exacerbation patterns in the second year. We drew the calibration plots for the entire sample, and by exacerbation history. Additionally, we used model-based receiver operating characteristic (mROC) curves for the primary outcome for statistical inference for model calibration without smoothing or grouping the data.^21^ To compare the calibration performance of different models, we used the integrated calibration index (ICI), a single summary of calibration (the lower the ICI the better), with bootstrap for generating confidence intervals (CI) and *p*-values when comparing difference in ICI between competing models.^22^

#### Model discrimination

To evaluate the discriminatory performance of ACCEPT 2·0, we created the standard ROC plots and calculated the AUC.^23^ To account for patients’ variable follow-up time, we used time-dependent (at year one) ROC and AUC^24^^,^^25^ for all outcomes. To compare prediction models, we used non-parametric tests specially designed for comparing time-dependent ROC curves and AUCs.^25^^,^^26^

#### Net benefit

We conducted a decision curve analysis for ACCEPT 2·0, ACCEPT 1·0, and exacerbation history alone. The decision curve quantifies the expected net benefit of using a risk prediction model or classification algorithm compared with alternative decisions (either giving treatment to all or to none).^27^ The basic principle underlying decision curves is that the risk threshold above which the treatment is recommended is informative of how the decision-maker weighs the relative benefit and harm of correct and incorrect decisions. This theoretical relationship is then used to derive the net benefit of the classification algorithm at any given cut-off value.^28^ One way of interpreting the decision curve at a given threshold is to compute the net difference in the proportion of false positives, which in turn can be expressed as the number of unnecessary interventions avoided for the model with better performance.^29^ In interpreting the decision curve, we chose the threshold of 65% as an exemplary threshold as a previous analysis has demonstrated that the current definition of ‘frequent exacerbator’ status implicitly puts a threshold for 12-month exacerbation risk around this value.^30^

All the analysis was done in R 4·0·2^31^ and SAS 9·4 (SAS Institute, Cary NC). The R packages and the functions used for different analyses is provided in Section 9 of the Supplementary Material.

### Role of the funding source

The funder had no role in the study design, data collection, analysis, interpretation, writing of the report, and in the decision to submit the paper for publication.

## Results

### Participants

Table 1 summarises the baseline characteristics and outcomes in the recalibration (ECLIPSE) and validation (TORCH) samples. The recalibration sample contained data from 1,803 individuals (mean age 63·3 years, 65·2% male), contributing 1,646 moderate and 471 severe exacerbations. The details of comparison between the predictors and outcomes of the development dataset are available in Supplementary Material (Table SM1). The external validation sample included 1,091 patients (mean age 65·5 years, 77·2% male), contributing 886 moderate and 188 severe exacerbations. There were 284 (12·9%) patients with short follow-up (<0·3 year) and 109 (5·0%) patients with missing outcomes in the recalibration dataset, and 433 (28·4%) patients with short follow-up in the external validation dataset that were excluded in our analyses. There was no difference in predicted risks between the excluded and included patients (Supplementary Material - Figure SM3). The observed rates of moderate/severe exacerbations were 1·20 and 1·02 events/year in the recalibration and validation samples, respectively. The corresponding rates for severe exacerbations were 0·27 and 0·18, respectively.

**Table 1:** Baseline characteristics and outcomes in the recalibration and validation samples.

*LAMA use is based on the imputed data for the validation study.

BMI, body mass index; COPD, chronic obstructive pulmonary disease; FEV_1_, forced expiratory volume in 1 second; ICS, inhaled corticosteroids; LABA, long-acting β agonist; LAMA, long-acting muscarinic receptor antagonist; SGRQ, St George's Respiratory Questionnaire score; SD, standard deviation.

### Model recalibration

**Figure 2:**
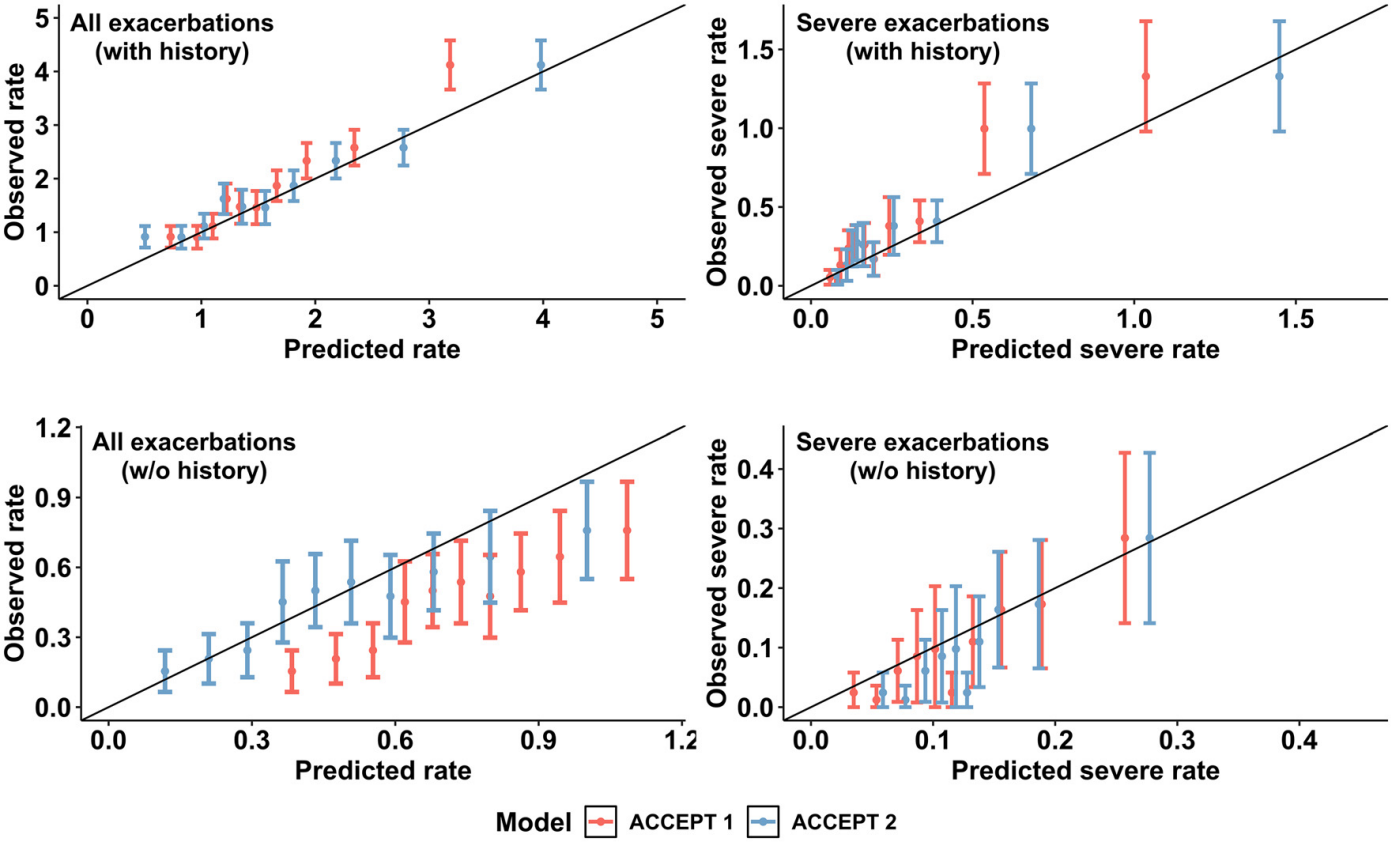
Calibration plots of ACCEPT 1·0 (red) and ACCEPT 2·0 (blue) along with the 95% confidence interval of the observed rate per deciles in Evaluation of COPD Longitudinally to Identify Predictive Surrogate End-points (ECLIPSE) study in patients with (top) and without (bottom) history of exacerbations. ACCEPT, Acute COPD Exacerbation Prediction Tool; COPD, chronic obstructive pulmonary disease; w/o, without.

Figure 2 shows the calibration plots before (ACCEPT 1·0) and after (ACCEPT 2·0) recalibration for moderate/severe (left) and severe (right) exacerbations in patients with (top) and without (bottom) exacerbation history in the ECLIPSE cohort. The most obvious improvement in calibration occurred for moderate/severe exacerbations among patients without an exacerbation history (bottom-left panel; difference in ICI between ACCEPT 1·0 and 2·0 = 0·057; *p*-value = 0·003). Results for the reduced ACCEPT 2·0 models generally followed the same pattern. Figure SM1 and Tables SM6-SM9 in the Supplementary Material provides visual illustration and parameter estimates of the fitted splines. A comparison between predicted and observed rates among different patients’ subgroups is provided in Supplementary Material - Table SM11.

### External validation

#### Calibration

**Figure 3:**
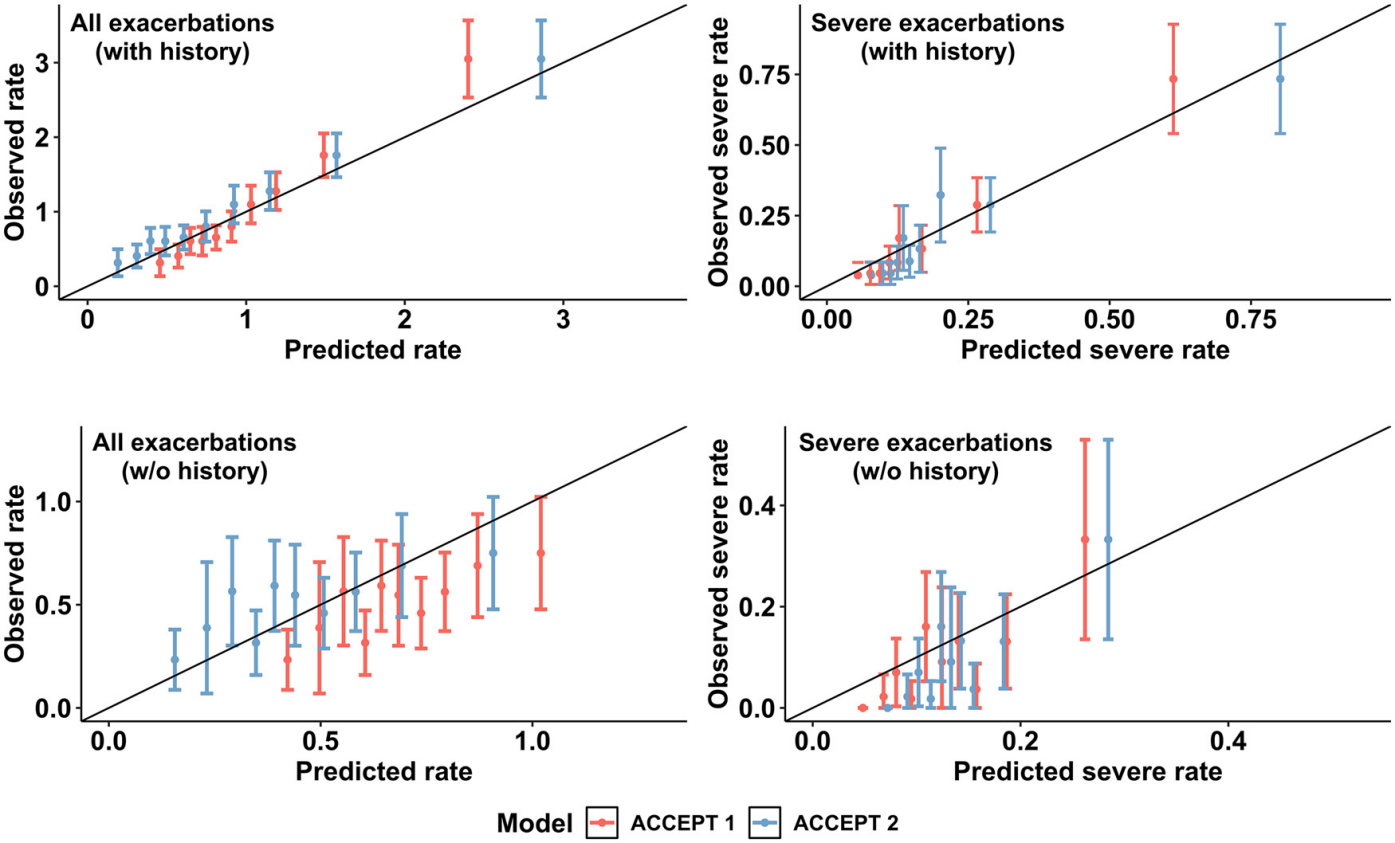
Calibration plots of ACCEPT 2·0 along with the 95% confidence interval of the observed rate per deciles in the Towards a Revolution in COPD Health (TORCH) study in patients with (top) and without (bottom) history of exacerbations. ACCEPT, Acute COPD Exacerbation Prediction Tool; COPD, chronic obstructive pulmonary disease; w/o, without.

Figure 3 presents the calibration plots before (ACCEPT 1·0) and after (ACCEPT 2·0) recalibration for moderate/severe (left) and severe (right) exacerbations among patients with (top) and without exacerbation (bottom) history in the TORCH sample. The model was generally well calibrated in the external sample for both groups and by exacerbation severity (calibration-in-the-large [average predicted and observed rate difference]: 0·13). Again, ACCEPT 2·0 showed improvement in calibration for moderate/severe exacerbations in patients without an exacerbation history (bottom-left panel; difference in ICI = 0·042; *p*-value = 0·034). Additionally, Figure SM2 of Supplementary Material shows the mROC and ROC curves for the primary outcome (≥2 moderate or ≥1 severe events). The mROC test for model miscalibration was not significant (*p* = 0·38). The improvement in the ICI of ACCEPT 2·0 compared with ACCEPT 1·0 was significant for both the primary outcome and for predicting any moderate/severe exacerbations (change in ICI = 0·038 [*p* = 0·009] and ICI = 0·040 [*p* = 0·028], respectively).

In sensitivity analyses, the calibration of both versions of ACCEPT were similar in the treatment arms; this was also the case in the sensitivity analysis where LAMA was set to zero in TORCH. See Supplementary Material for the ICI results of the two ACCEPT models for patients with different exacerbation history (Section 5), the calibration plots of ACCEPT models on treatment arms of TORCH study (Section 8), the calibration plots of ACCEPT models where LAMA was set to zero for all patients (Section 9), and a comparison between predicted and observed rates among different patients’ subgroups (Section 7).

### Discrimination

For the primary outcome, ACCEPT 2·0 had an AUC of 0·76 (95%CI: 0·72, 0·79) compared to an AUC of 0·75 (95%CI: 0·72, 0·79; *p* = 0.026) for ACCEPT 1·0. When separating exacerbations by type, the AUC for ACCEPT 2·0 was 0·73 (95%CI: 0·71, 0·76) for predicting ≥1 moderate/severe, and 0·76 (95%CI: 0·72, 0·81) for predicting ≥1 severe exacerbation. The corresponding AUC values for the history of exacerbations were 0·67 (95%CI: 0·64, 0·70; p<0·001) and 0·61 (95%CI: 0·57, 0·65; p<0·001), respectively. Figure SM2 of Supplementary Material shows the ROC curves and AUCs.

### Net benefit

**Figure 4:**
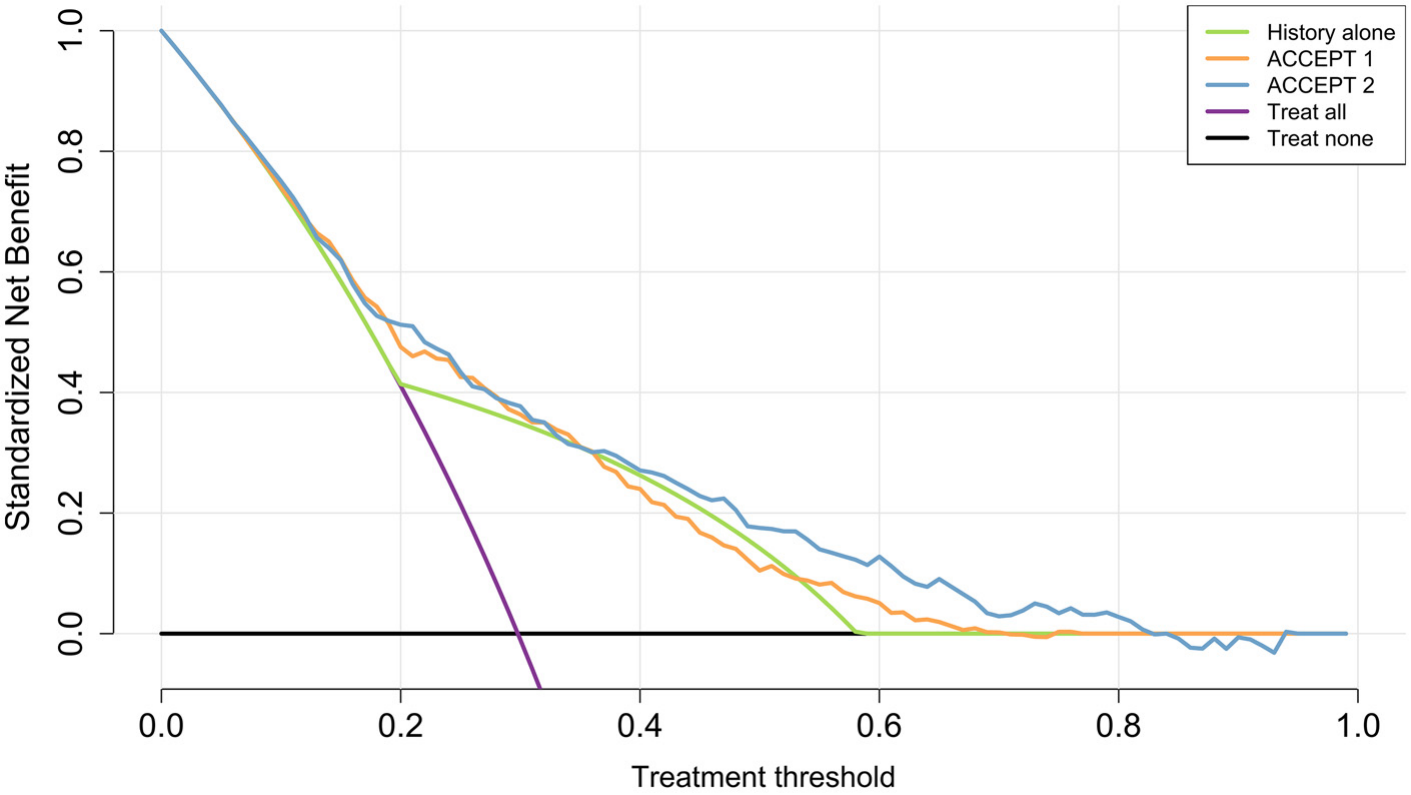
Decision curves to predict frequent exacerbators for the model predictions and history in the Towards a Revolution in COPD Health (TORCH) study. Net benefit of history alone at threshold of 65%: 0%; Net benefit of ACCEPT 1·0 at threshold of 65%: 1.9%; Net benefit of ACCEPT 2·0 at threshold of 65%: 9.1%. ACCEPT, Acute COPD Exacerbation Prediction Tool; COPD, chronic obstructive pulmonary disease. Net benefit of ACCEPT 2·0 at threshold of 65%: 9.1%.

Figure 4 shows the decision curves before (ACCEPT 1·0) and after (ACCEPT 2·0) recalibration for predicting the primary outcome. For thresholds between 0·16 and 0·81, ACCEPT 2·0 provided superior clinical utility compared with exacerbation history alone, treating no one, or treating all. Within this range, only in a narrow band around the threshold probability of 35% did ACCEPT 2·0 and exacerbation history provide similar clinical utility. Additionally, ACCEPT 2·0 outperformed ACCEPT 1·0 in net benefit for all thresholds between 0·30 and 0·81. At a threshold of 65%, the use of ACCEPT 2·0 was equivalent to a strategy that reduced the number of unnecessary treatment by about 10 per 100 decisions without missing treatment for any patient who would experience the primary outcome. This number for ACCEPT 1·0 and history alone were 2 and 0 per 100 decisions, respectively. In sensitivity analyses, the decision curves of both versions of ACCEPT were similar in the entire data of the TORCH study (Section 8 in Supplementary Material).

### Simplified models and secondary outcomes

Table 2 reports the AUC of full models and reduced versions for different outcomes. The difference between the AUC of the full model and its reduced versions was minimal and non-significant for the primary outcome (≥2 moderate or ≥1 severe exacerbations in the next 12 months - ΔAUC < 0·006; *p*-value > 0·34), or predicting any moderate/severe exacerbations in the next 12 months (ΔAUC < 0·011; *p*-value > 0·18). However, for predicting any severe exacerbations in the next 12 months, the full model outperformed the reduced models. On the other hand, the calibration and net benefit of the reduced versions of ACCEPT 2·0 were close to those of the full model (see Figure SM2 of Supplementary Material). The overall discriminative power and net benefit of the reduced models were better than the history of exacerbations alone. The net benefit of the simplified models was similar to that of the full model for different thresholds for the primary outcome. Figure SM2 of Supplementary Material display the ROC and decision curves of the simplified versions of ACCEPT 2·0.

**Table 2:** Time-dependent area under the curve (at year 1) of different models for prediction outcomes in the external validation sample (Towards a Revolution in Chronic Obstructive Pulmonary Disease Health study).

Model	≥2 moderate or ≥1 severe exacerbator	≥1 moderate/severe exacerbation	≥1 severe exacerbation
**Full model, AUC (95% CI)**	0·756 (0·724, 0·789)	0·735 (0·706, 0·765)	0·764 (0·722, 0·807)
**No COPD medications**	0·762 (0·730, 0·794)	0·732 (0·702, 0·762)	0·766 (0·725, 0·807)
	*p*-value = 0·340	*p*-value = 0·568	*p*-value = 0·819
**No SGRQ score**	0·759 (0·725, 0·792)	0·733 (0·703, 0·763)	0·744 (0·699, 0·789)
	*p*-value = 0·662	*p*-value = 0·609	*p*-value = 0·099
**No COPD medications and no SGRQ score**	0·761 (0·728, 0·794)	0·724 (0·694, 0·755)	0·754 (0·710, 0·798)
	*p*-value = 0·550	*p*-value = 0·186	*p*-value = 0·530

COPD, chronic obstructive pulmonary disease; SGRQ, St George's Respiratory Questionnaire; COPD, chronic obstructive pulmonary disease; AUC, area under the curve; CI, confidence interval.

*p*-values are to compare reduced models with the full model for each outcome.

## Discussion

We updated a previously developed exacerbation risk prediction model (ACCEPT 1·0) to accomplish two main goals: 1) to make better predictions of exacerbation risk for patients without an exacerbation in the previous 12 months, and 2) to investigate whether more parsimonious versions of ACCEPT would provide comparable performance. We showed that the resulting ACCEPT 2·0 can predict risk and severity of exacerbations with high accuracy, regardless of exacerbation history. We also showed that ACCEPT 2·0 is likely to provide higher clinical utility compared with the use of exacerbation history alone at a wide range of risk thresholds. We have updated our publicly available Web app for ACCEPT, which is available at: https://resp.core.ubc.ca/ipress/accept, as well as the accept R package which is available on the comprehensive R archive network (https://CRAN.R-project.org).

Additionally, we showed that the reduction in AUC and net benefit in simplified models was minimal for the primary outcome (predicting ≥2 moderate or ≥1 severe exacerbations) and for predicting any moderate/severe exacerbations, but more substantial for predicting any severe exacerbations (0·020 reduction for the model without SGRQ score and medications). In creating parsimonious models, removing the SGRQ score as a predictor had a minimal impact on the overall performance of the model in predicting the primary outcome. Symptom burden (e.g., degree of breathlessness) is a known predictor of exacerbation risk.^32^ However, it might be the case that other predictor scores (such as COPD treatments) are acting as surrogates for patient symptoms. Of note, one can use the simpler COPD Assessment Test score in lieu of SGRQ in ACCEPT, but even this score is not routinely available in EHR. As such, the version of ACCEPT without a symptom score can have applicability in the implementation of risk stratification within EHR systems. Another application of this tool is its potential for prognostic enrichment of COPD trials.^33^ Many clinical trials in COPD target exacerbations as their primary endpoint. However, due to the stochastic nature of exacerbations, demonstrating treatment effect often requires large sample sizes. A tool like ACCEPT can be used to recruit individuals who are at high risk of events (e.g., based on specifying a threshold on predicted exacerbation risk) to achieve the same statistical power at lower sample sizes.^33^

A systematic review of exacerbation risk prediction models in 2016 concluded that, of the 27 models meeting the inclusion criteria, none were ready for clinical use.^13^ The most salient limitation has been poor replication of models in independent samples. Yet, such external validation is key to the credibility and acceptability of risk predictions in clinical practice. A large systematic review of 228 studies developing, validating, or updating a prediction model in COPD showed that these models were externally validated on average 0·09 times,^34^ whereas a similar review for cardiovascular risk prediction models reported an average of 1·30 external validations per model.^35^ Addressing a previously identified calibration issue with ACCEPT 1·0, updating the model, and conducting a new external validation task (in particular showing the higher net benefit of ACCEPT 2·0 compared with exacerbation history alone), can provide reassurance and motivation for using this tool for risk stratification in COPD.

This study had several strengths. Updating existing models, rather than developing new ones, is a preferred approach for facilitating risk prediction across different populations and settings.^36^ We had access to individual-level data for multiple studies in heterogenous settings (e.g., both clinical trials and observational studies) to develop, recalibrate, and validate the model. In recalibrating the model, we used non-parametric spline methods that flexibly adjust for miscalibration without requiring refitting the original model. Spline methods are generally more parsimonious than refitting all regression coefficients of the model, and thus are less likely to overfit the data. Further, most exacerbation risk scoring tools are based on logistic regression models that predict the binary outcome of whether any exacerbation will occur during a pre-defined time window.^17^^,^^37^ In comparison, a unique feature of ACCEPT is its ability to make nuanced predictions of the future rates of both moderate and severe exacerbations, which can be converted to predicting the likelihood of any combination of exacerbations. In particular, we were able to focus on predicting the occurrence of ≥2 moderate or ≥1 severe exacerbations in the next 12 months as the primary outcome, which is contemporarily used to define patients who are eligible for the escalation of therapies.

The limitations of this study should be acknowledged. Several candidate predictors of exacerbations were not available in the underlying datasets, such as blood biomarkers (e.g., eosinophils) and comorbidities, other than cardiovascular risk which was proxied by statin use. The clinical trials underlying ACCEPT 1·0 excluded patients with a history of asthma, never-smokers, and patients younger than 40 years of age. As such, the accuracy of predictions in these subgroups are not known. Further, the external validation sample used in this study consisted of predominantly male individuals, and is based on relatively old data. Further examination of ACCEPT 2·0 in more diverse and contemporary populations is therefore warranted. Moreover, a recent study has documented international differences in the frequency of COPD exacerbations even among patients with the same exacerbation history.^38^ It is not clear to what extent the presence of multiple other predictors could explain away such variability. In general, it is likely that prediction models of exacerbations need to be recalibrated to their specific settings (e.g., country, type of care [primary, secondary, or tertiary], and socioeconomic status), as has been demonstrated recently for cardiovascular risk prediction models.^39^ Compared with exacerbation history alone, implementing ACCEPT requires knowledge of several variables and its calculations involves sophisticated non-linear regression model. This makes manual use of the model difficult. However, the increasing accessibility to computers and EHRs at point of care should mitigate these concerns across many settings.

The central role of exacerbation history for risk stratification in the contemporary management of COPD is based on the notion that the best predictor of future exacerbation risk is a previous history of exacerbations.^3^ Nevertheless, due to the stochastic nature of individual exacerbations, exacerbation patterns change from one year to another, so much so that the suitability of exacerbation history alone for informing treatment is questionable.^40^ The pathophysiology of exacerbations is complex and is likely affected by many intrinsic and extrinsic factors. As such, several other predictors in combination can significantly improve the predictability of future exacerbations over and beyond exacerbation history alone, as our results importantly demonstrate. The resulting ACCEPT risk score shows promises in conferring significantly higher clinical utility over exacerbation history alone. The next step is to validate and potentially update models of this type in diverse subgroups of patients, and to conduct real-world impact studies that directly compare the effect of using different risk stratification methods on patient care and outcomes.

## Contributors

MS and DS conceptualized the study. AS, AA, and TYL developed the data analysis plan. DS provided access to the data. AS performed data analysis. JKH re-ran the data analysis. AS and MS wrote the first version of the manuscript. AA developed the Web application. All authors critically revised the manuscript and approved the final version. MS had full access to the data and is the guarantor of the study.

## Data sharing statement

The data used for this study were obtained under license. While the data are not publicly available, interested parties can apply to the pertinent data providers for data access requests. The code used for data analyses is available from https://github.com/resplab/papercode/tree/main/ACCEPT2.0.

## Declaration of interests

DS has received honorariums for COPD lectures sponsored by AstraZeneca, GSK, and Boehringer Ingelheim. All other authors declare no actual or perceived conflicts of interest.

## Appendix Supplementary materials

Image, application 1

Image, application 2
