# Supplementary material for: ACCEPT 2·0: Recalibrating and externally validating the Acute COPD exacerbation prediction tool (ACCEPT)
Source: eClinicalMedicine. 2022 Jul 22;51:101574. doi: 10.1016/j.eclinm.2022.101574 (PMC9309408; doi:10.1016/j.eclinm.2022.101574)
Supplement: Supplementary file 1 [file mmc1.docx]

Supplementary Material for “ACCEPT 2·0: Recalibrating and Externally Validating the ACute COPD Exacerbation Prediction Tool (ACCEPT)”

1. **Development (internal) and validation (external) data**

**Table SM1.** Study details of the data sources

| Data source | Design | Exposures | Follow-up | Inclusion criteria | Exclusion criteria |
| --- | --- | --- | --- | --- | --- |
| Internal |  |  |  |  |  |
| MACRO | RCT | Azithromycin | 1 year | >40 yr. Clinical diagnosis of COPD. >10 pack-years of smoking. O2 or systemic glucocorticoids therapy in the last yr. Hospitalization or ER visit. | Asthma. Exacerbation in the last month. HR above 100 /min. QTC >450ms. QTC prolonging or TdP-related medication except for amlodipine. Hearing impairment. |
| STATCOPE | RCT | Simvastatin | ~2 years | 40-80 yrs. Clinical diagnosis of COPD. >10 pack-years of smoking. O2 OR systemic glucocorticoids OR antibiotics therapy OR hospitalization OR ER visit in the last yr. | Asthma. Receiving statins, or should have received statins. On drugs that contradicted with statins. Unable to take statins. Active liver disease, alcoholism, or allergy. |
| OPTIMAL | RCT | Tiotropium with Salmeterol or Fluticasone-Salmeterol | 1 year | >35 yr. Clinical diagnosis of COPD. >10 pack-years of smoking. Exacerbation requiring systemic glucocorticoids OR antibiotics therapy in the last yr. | Asthma <40 yrs. CHF with persistent severe LVD. Oral prednisone. Intolerance to tiotropium, salmeterol, or fluticasone-salmeterol. Glaucoma. Urinary tract obstruction. Lung transplant or volume reduction. Diffuse bilateral bronchiectasis. Pregnancy or breastfeeding. |
| ECLIPSE | Cohort |  | 3 years | 40-75 yrs. Clinical diagnosis of COPD. >10 pack-years of smoking. | Respiratory disorders other than COPD. Reported exacerbation in the past month. Significant inflammatory disease. |
| External |  |  |  |  |  |
| TORCH | RCT | Salmeterol and Fluticasone alone or combined | 3 years | 40-80 yrs. Clinical diagnosis of COPD. >10 pack-years of smoking. FEV1≤60% pred., ≤10% reversibility in pred. FEV1, and FEV1/FVC≤70%. | Asthma or respiratory disorders other than COPD. Lung-volume reduction surgery. Lung transplant. LTOT>12h.day^-1^. Long-term oral corticosteroid therapy. |

1. **ACCEPT parameter estimates**

Since we removed patients with short follow-up time (less than 0·3 years), our model coefficient estimates were slightly different from those of ACCEPT (Table 2 of ACCEPT). However, the overall impact of the coefficients (direction and significance) on both outcomes were the same for most predictors. We observed a similar result for the parameters of the random effects.

**Table SM2.** Coefficient estimates for the joint rate-severity model (development model) – full model (ACCEPT)

| **Predictor** | **Rate component** | | **Severity component** | |
| --- | --- | --- | --- | --- |
|  | **HR (95% CI)** | **p-value** | **OR (95% CI)** | **p-value** |
| **Male** | 0·831 (0·74, 0·93) | 0·001 | 1·167 (1·13, 2·07) | 0·006 |
| **Age (per 10-year)** | 0·992 (0·92, 1·06) | 0·82 | 1·102 (0·91, 1·33) | 0·326 |
| **Current smoker** | 0·83 (0·72, 0·96) | 0·011 | 1·212 (1·05, 2·22) | 0·028 |
| **Oxygen therapy last year** | 1·129 (0·99, 1·29) | 0·071 | 1·202 (1·2, 2·48) | 0·003 |
| **FEV1 (% predicted)** | 0·572 (0·38, 0·86) | 0·007 | 1·839 (0·13, 1·47) | 0·184 |
| **SGRQ score (per 10-unit)** | 1·112 (1·07, 1·15) | 0·000 | 1·05 (1·09, 1·31) | 0·000 |
| **CV-indicated statins** | 1·146 (1, 1·31) | 0·042 | 1·201 (0·88, 1·81) | 0·205 |
| **BMI (per 10-unit)** | 0·875 (0·8, 0·96) | 0·005 | 1·146 (0·69, 1·19) | 0·479 |
| **LAMA** | 1·183 (1·05, 1·33) | 0·007 | 1·182 (0·61, 1·18) | 0·328 |
| **LABA** | 1·12 (0·97, 1·29) | 0·109 | 1·209 (0·73, 1·53) | 0·773 |
| **ICS** | 1·322 (1·15, 1·52) | 0·000 | 1·207 (0·9, 1·89) | 0·155 |
| **Random effects parameters** | | | | |
| **Variance** | 0·685 (0·58, 0·79) | 0·000 | 2·249 (1·48, 3·02) | 0·000 |
| **Covariance** | 0·088 (-0·13, 0·31) | 0·43 |  | |

**Table SM3.** Coefficient estimates for the joint rate-severity model (development model) – No COPD medications

| **Predictor** | **Rate component** | | **Severity component** | |
| --- | --- | --- | --- | --- |
|  | **HR (95% CI)** | **p-value** | **OR (95% CI)** | **p-value** |
| **Male** | 0·832 (0·74, 0·93) | 0·001 | 1·167 (1·13, 2·08) | 0·006 |
| **Age (per 10-year)** | 0·995 (0·93, 1·07) | 0·89 | 1·103 (0·91, 1·33) | 0·32 |
| **Current smoker** | 0·808 (0·7, 0·93) | 0·004 | 1·212 (1·06, 2·25) | 0·024 |
| **Oxygen therapy last year** | 1·148 (1·01, 1·31) | 0·038 | 1·202 (1·2, 2·46) | 0·003 |
| **FEV1 (% predicted)** | 0·499 (0·33, 0·75) | 0·001 | 1·836 (0·14, 1·48) | 0·188 |
| **SGRQ score (per 10-unit)** | 1·118 (1·08, 1·16) | 0·000 | 1·05 (1·09, 1·31) | 0·000 |
| **CV-indicated statins** | 1·201 (1·06, 1·37) | 0·006 | 1·204 (0·9, 1·86) | 0·169 |
| **BMI (per 10-unit)** | 0·874 (0·8, 0·96) | 0·004 | 1·146 (0·7, 1·2) | 0·543 |
| **Random effects parameters** | | | | |
| **Variance** | 0·708 (0·60, 0·81) | 0·000 | 2·275 (1·53, 3·02) | 0·000 |
| **Covariance** | 0·092 (-0·14, 0·32) | 0·43 |  | |

**Table SM4.** Coefficient estimates for the joint rate-severity model (development model) – No SGRQ

| **Predictor** | **Rate component** | | **Severity component** | |
| --- | --- | --- | --- | --- |
|  | **HR (95% CI)** | **p-value** | **OR (95% CI)** | **p-value** |
| **Male** | 0·836 (0·75, 0·93) | 0·002 | 1·169 (1·16, 2·13) | 0·004 |
| **Age (per 10-year)** | 0·96 (0·89, 1·03) | 0·261 | 1·102 (0·86, 1·26) | 0·692 |
| **Current smoker** | 0·873 (0·76, 1·01) | 0·062 | 1·214 (1·14, 2·44) | 0·009 |
| **Oxygen therapy last year** | 1·169 (1·02, 1·33) | 0·021 | 1·202 (1·28, 2·62) | 0·001 |
| **FEV1 (% predicted)** | 0·456 (0·31, 0·68) | 0·000 | 1·84 (0·08, 0·91) | 0·035 |
| **CV-indicated statins** | 1·178 (1·03, 1·35) | 0·015 | 1·203 (0·93, 1·92) | 0·119 |
| **BMI (per 10-unit)** | 0·897 (0·82, 0·99) | 0·023 | 1·148 (0·71, 1·22) | 0·622 |
| **LAMA** | 1·198 (1·06, 1·35) | 0·004 | 1·185 (0·62, 1·2) | 0·367 |
| **LABA** | 1·098 (0·95, 1·26) | 0·193 | 1·209 (0·7, 1·48) | 0·921 |
| **ICS** | 1·357 (1·18, 1·56) | 0·000 | 1·208 (0·93, 1·96) | 0·112 |
| **Random effects parameters** | | | | |
| **Variance** | 0·716 (0·61, 0·82) | 0·000 | 2·330 (1·57, 3·09) | 0·000 |
| **Covariance** | 0·146 (-0·08, 0·38) | 0·21 |  | |

**Table SM5.** Coefficient estimates for the joint rate-severity model (development model) – No SGRQ & no COPD medications

| **Predictor** | **Rate component** | | **Severity component** | |
| --- | --- | --- | --- | --- |
|  | **HR (95% CI)** | **p-value** | **OR (95% CI)** | **p-value** |
| **Male** | 0·836 (0·75, 0·94) | 0·002 | 1·169 (1·15, 2·13) | 0·004 |
| **Age (per 10-year)** | 0·961 (0·89, 1·03) | 0·279 | 1·102 (0·86, 1·26) | 0·664 |
| **Current smoker** | 0·851 (0·74, 0·98) | 0·027 | 1·215 (1·15, 2·48) | 0·007 |
| **Oxygen therapy last year** | 1·194 (1·05, 1·36) | 0·008 | 1·202 (1·28, 2·63) | 0·001 |
| **FEV1 (% predicted)** | 0·395 (0·26, 0·59) | 0 | 1·84 (0·08, 0·89) | 0·032 |
| **CV-indicated statins** | 1·241 (1·09, 1·42) | 0·001 | 1·206 (0·96, 2) | 0·085 |
| **BMI (per 10-unit)** | 0·895 (0·81, 0·98) | 0·02 | 1·148 (0·72, 1·23) | 0·663 |
| **Random effects parameters** | | | | |
| **Variance** | 0·742 (0·63, 0·85) | 0·000 | 2·378 (1·60, 3·16) | 0·000 |
| **Covariance** | 0·156 (-0·08, 0·40) | 0·20 |  | |

1. **Multivariate adaptive regression splines (MARS) model fit**

The MARS model involves a set of simple piecewise functions that characterize the data and using them in aggregate to predict the output. In a sense, the MARS model is an ensemble of simple functions.

The form of our MARS regression models is

$$Y_{i}=\beta_{0}+\sum_{k=1}^{4} \beta_{k}B_{k}(X_{i})$$

where $Y_{i}$ and $X_{i}$ respectively are the observed and predicted exacerbation rate for i^th^ patient, $\beta_{i}$’s are the MARS regression coefficients, and $B_{k}$’s are the natural cubic spline basis functions. Practically, a cubic spline model breaks up the data to different regions (by using predictor’s quantiles – aka “knots”) and fits a cubic multinomial model in each region. By adding some constrains on the model parameters, it results in a smooth fitted curve across different regions. To avoid overfitting, we use one of the simplest form of MARS model by using cubic spline basis functions (cubic multinomial function for each region – the most common basis functions) and setting the number of knots to 3 (i.e., four regions – minimum number of knots in a natural cubic spline model).

Figure SM1 illustrates the relationship between the ACCEPT 1 predicted rates (x-axis) and the observed rates (y-axis). The MARS model (the orange line) resulted in a nearly-linear recalibration of predicted risks of all exacerbations from ACCEPT (left panel), and appeared less linear for severe AECOPDs (orange line in right panel). ACCEPT 2 uses these smoothed fitted curves based on the relationship between ACCEPT 1 predictions and the observations to obtain more calibrated rates at the end.

**Figure SM1**. Percentile calibration plot of the MARS model for all (left) and severe (right) exacerbations.


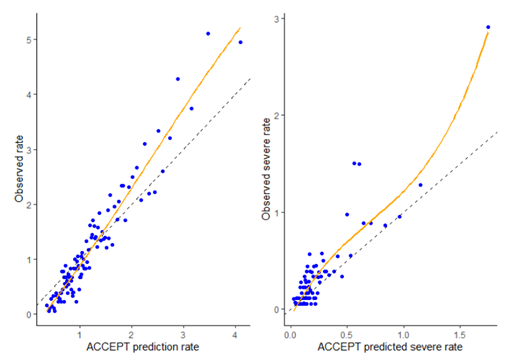


**Example from ACCEPT 1:**

We borrow the example below from the supplementary materials of ACCEPT 1 (Table S1):


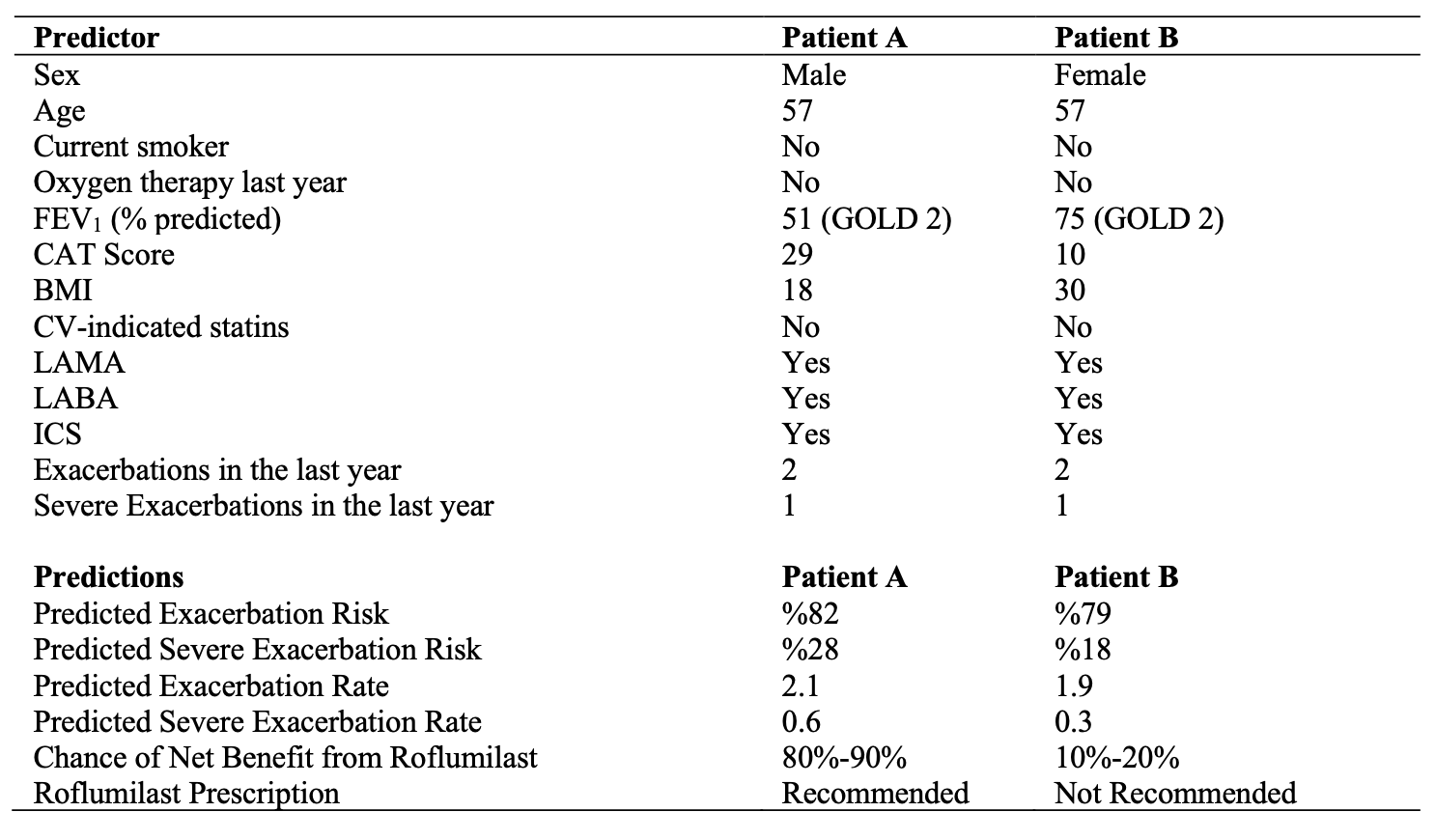


The predicted risk for these two patients from ACCEPT 1 were 2.1 and 1.9. Since the knots of the full model MARS model were 0.714, 0.991, and 1.465 (Table SM6 in the next section), the ACCEPT 1 predicted value of both of these two patients fall in the same region (i.e., greater than 3^rd^ knots). Therefore, the same set of basis functions in terms of a cubic polynomial function of the difference between their predicted rate and the 3^rd^ knot will apply on both to produce ACCEPT 2 predicted rates. To see the generated cubic polynomial function based on the fitted MARS model, one can use R package SplineUtils available here. One can pass aa fitted spline model to the function *RegBsplineAsPiecePoly* from this package to produce such polynomial functions. Finally, the ACCEPT 2 predicted rates of these two patients will be 2.43 and 2.15, respectively.

1. **MARS model parameter estimates**

**Table SM6.** Coefficient estimates of the MARS models for all and severe exacerbations (updating) – full model

| **Predictor** | **Rate of all exacerbations** | | **Rate of severe exacerbations** | |
| --- | --- | --- | --- | --- |
|  | **Est (95% CI)** | **p-value** | **Est (95% CI)** | **p-value** |
| **Intercept** | 16·01 (-14·01, 17·93) | 0·000 | 7·11 (5·65, 8·56) | 0·000 |
| **ns(prediction, df = 4) 1** | -12·48 (-14·04, -10·91) | 0·000 | -5·47 (-6·61, -4·32) | 0·000 |
| **ns(prediction, df = 4) 2** | -15·05 (-16·93, -13·17) | 0·000 | -6·95 (-8·39, -5·51) | 0·000 |
| **ns(prediction, df = 4) 3** | -4·40 (-5·45, -3·34) | 0·000 | -1·89 (-2·63, -1·15) | 0·000 |
| **ns(prediction, df = 4) 4** | -31·87 (-35·96, -27·79) | 0·000 | -14·22 (-17·32, -11·13) | 0·000 |
| **Knot 1 (25%)** | 0·714 |  | 0·110 |  |
| **Knot 2 (50%)** | 0·991 |  | 0·155 |  |
| **Knot 3 (75%)** | 1·465 |  | 0·244 |  |

**Table SM7.** Coefficient estimates of the MARS models for all and severe exacerbations (updating) – No COPD medications

| **Predictor** | **Rate of all exacerbations** | | **Rate of severe exacerbations** | |
| --- | --- | --- | --- | --- |
|  | **Est (95% CI)** | **p-value** | **Est (95% CI)** | **p-value** |
| **Intercept** | 15·42 (13·53, 17·31) | 0·000 | 6·82 (5·45, 8·20) | 0·000 |
| **ns(prediction, df = 4) 1** | -12·17 (-13·72, -10·61) | 0·000 | -5·31 (-6·40, -4·21) | 0·000 |
| **ns(prediction, df = 4) 2** | -14·39 (-16·25, -12·53) | 0·000 | -6·66 (-8·03, -5·30) | 0·000 |
| **ns(prediction, df = 4) 3** | -4·16 (-5·21, -3·12) | 0·000 | -1·79 (-2·48, -1·09) | 0·000 |
| **ns(prediction, df = 4) 4** | -30·53 (-34·57, -26·49) | 0·000 | -13·61 (-16·54, -10·05) | 0·000 |
| **Knot 1 (25%)** | 0·687 |  | 0·102 |  |
| **Knot 2 (50%)** | 0·917 |  | 0·142 |  |
| **Knot 3 (75%)** | 1·333 |  | 0·216 |  |

**Table SM8.** Coefficient estimates of the MARS models for all and severe exacerbations (updating) – No SGRQ

| **Predictor** | **Rate of all exacerbations** | | **Rate of severe exacerbations** | |
| --- | --- | --- | --- | --- |
|  | **Est (95% CI)** | **p-value** | **Est (95% CI)** | **p-value** |
| **Intercept** | 15·85 (13·91, 17·79) | 0·000 | 9·87 (5·32, 8·01) | 0·000 |
| **ns(prediction, df = 4) 1** | -12·41 (-14·00, -10·82) | 0·000 | -9·61 (-6·18, -4·06) | 0·000 |
| **ns(prediction, df = 4) 2** | -14·83 (-16·74, -12·93) | 0·000 | -9·71 (-7·86, -5·19) | 0·000 |
| **ns(prediction, df = 4) 3** | -4·58 (-5·66, -3·50) | 0·000 | -4·87 (-2·33, -0·98) | 0·000 |
| **ns(prediction, df = 4) 4** | -31·49 (-35·64, -27·34) | 0·000 | -9·22 (-16·16, -10·42) | 0·000 |
| **Knot 1 (25%)** | 0·746 |  | 0·114 |  |
| **Knot 2 (50%)** | 0·987 |  | 0·149 |  |
| **Knot 3 (75%)** | 1·421 |  | 0·211 |  |

**Table SM9.** Coefficient estimates of the MARS models for all and severe exacerbations (updating) – No SGRQ & no COPD medications

| **Predictor** | **Rate of all exacerbations** | | **Rate of severe exacerbations** | |
| --- | --- | --- | --- | --- |
|  | **Est (95% CI)** | **p-value** | **Est (95% CI)** | **p-value** |
| **Intercept** | 15·36 (13·41, 17·32) | 0·000 | 6·36 (4·98, 7·73) | 0·000 |
| **ns(prediction, df = 4) 1** | -12·34 (-13·97, -10·71) | 0·000 | -4·93 (-6·02, -3·85) | 0·000 |
| **ns(prediction, df = 4) 2** | -14·27 (-16·19, -12·35) | 0·000 | -6·17 (-7·53, -4·81) | 0·000 |
| **ns(prediction, df = 4) 3** | -4·33 (-5·43, -3·24) | 0·000 | -1·77 (-2·47, -1·08) | 0·000 |
| **ns(prediction, df = 4) 4** | -30·29 (-34·44, -26·13) | 0·000 | -12·75 (-15·67, -9·82) | 0·000 |
| **Knot 1 (25%)** | 0·702 |  | 0·102 |  |
| **Knot 2 (50%)** | 0·913 |  | 0130 |  |
| **Knot 3 (75%)** | 1·316 |  | 0·183 |  |

1. **Integrated Calibration Index (ICI) of different models**

**Table SM10**. ΔICI (95% CI) of the full model comparing ACCEP 1 & 2 for different COPD events breakdown by exacerbation history on TORCH study.

| Model | High-risk exacerbator | Moderate/severe exacerbation | Severe exacerbation |
| --- | --- | --- | --- |
| All patients | 0·038  (0·018, 0·074) | 0·040  (0·006, 0·075) | 0·001  (-0·011, 0·004) |
|  | p-value=0·009 | p-value=0·028 | p-value=0·822 |
| Patients with history | 0·076  (0·059, 0·146) | 0·061  (0·037, 0·105) | 0·003  (-0·020, 0·007) |
|  | p-value=0·003 | p-value=0·004 | p-value=0·638 |
| Patients without history | 0·002  (-0·017, 0·022) | 0·042  (0·012, 0·104) | 0·011  (0·0001, 0·022) |
|  | p-value=0·875 | p-value=0·034 | p-value=0·046 |
| *COPD, chronic obstructive pulmonary disease; SGRQ, St George’s Respiratory Questionnaire.* | | | |

1. **Decision curve analysis (DCA) of different models**

***Figure SM2*** presents DCA (left panel) and ROC (right panel) curves of the high-risk exacerbator events predictions based on different versions of ACCEPT 2·0 in the external validation data (TORCH). Based on the DCA and ROC curves (including the C statistics), removing COPD medications and SGRQ predictors from the full model did not have negative impact on neither the clinical utility nor discriminative power of the model when the outcome was being a high-risk exacerbator. See Supplementary Materials (Table SM10) for the performance of different versions of ACCEPT 2·0 for other AECOPD events.

**Figure SM2**. DCA (left panel) and ROC (right panel) curves of high-risk exacerbator events predictions for different versions of ACCEPT 2 in TORCH study.


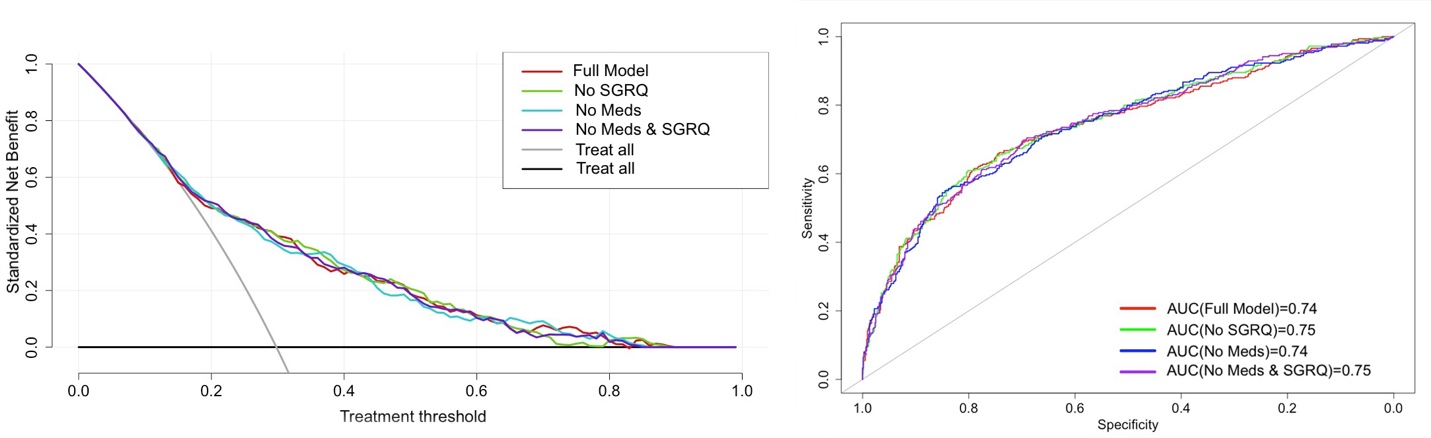


1. **Observed and predicted rates of exacerbations**

**Table SM11.** Observed and predicted rate of COPD moderate/severe and severe exacerbations in different subgroups of patients.

| Data | Subgroup | Outcome | Observed  rate | ACCEPT 1 | ACCEEPT 2 |
| --- | --- | --- | --- | --- | --- |
| ECLIPSE  (internal) | All | Moderate/severe | 1.19 | 1.19 | 1.16 |
|  |  | severe | 0.27 | 0.21 | 0.26 |
|  | With history | Moderate/severe | 1.81 | 1.59 | 1.72 |
|  |  | severe | 0.40 | 0.29 | 0.36 |
|  | W/O history | Moderate/severe | 0.46 | 0.71 | 0.50 |
|  |  | severe | 0.10 | 0.12 | 0.13 |
| TORCH  (external) | All | Moderate/severe | 1.02 | 1.01 | 0.91 |
|  |  | severe | 0.18 | 0.18 | 0.21 |
|  | With history | Moderate/severe | 1.60 | 1.36 | 1.39 |
|  |  | severe | 0.28 | 0.24 | 0.28 |
|  | W/O history | Moderate/severe | 0.50 | 0.70 | 0.47 |
|  |  | severe | 0.09 | 0.13 | 0.14 |

**Figure SM3.** Boxplot of predicted rates for patients with long follow-up and dropouts.


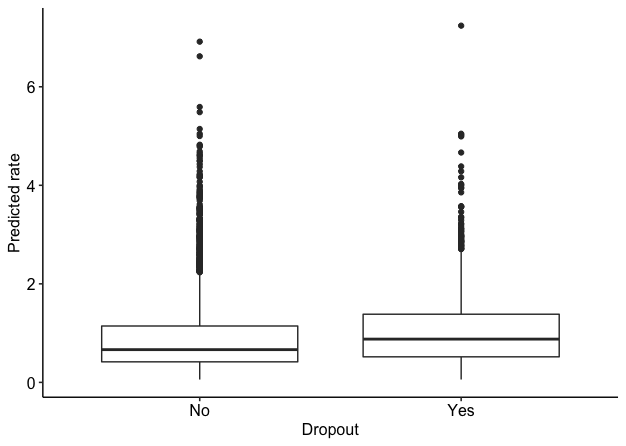


1. **Sensitivity analysis: treatment arms of TORCH study**

**Figure SM3**. Event: frequent exacerbator


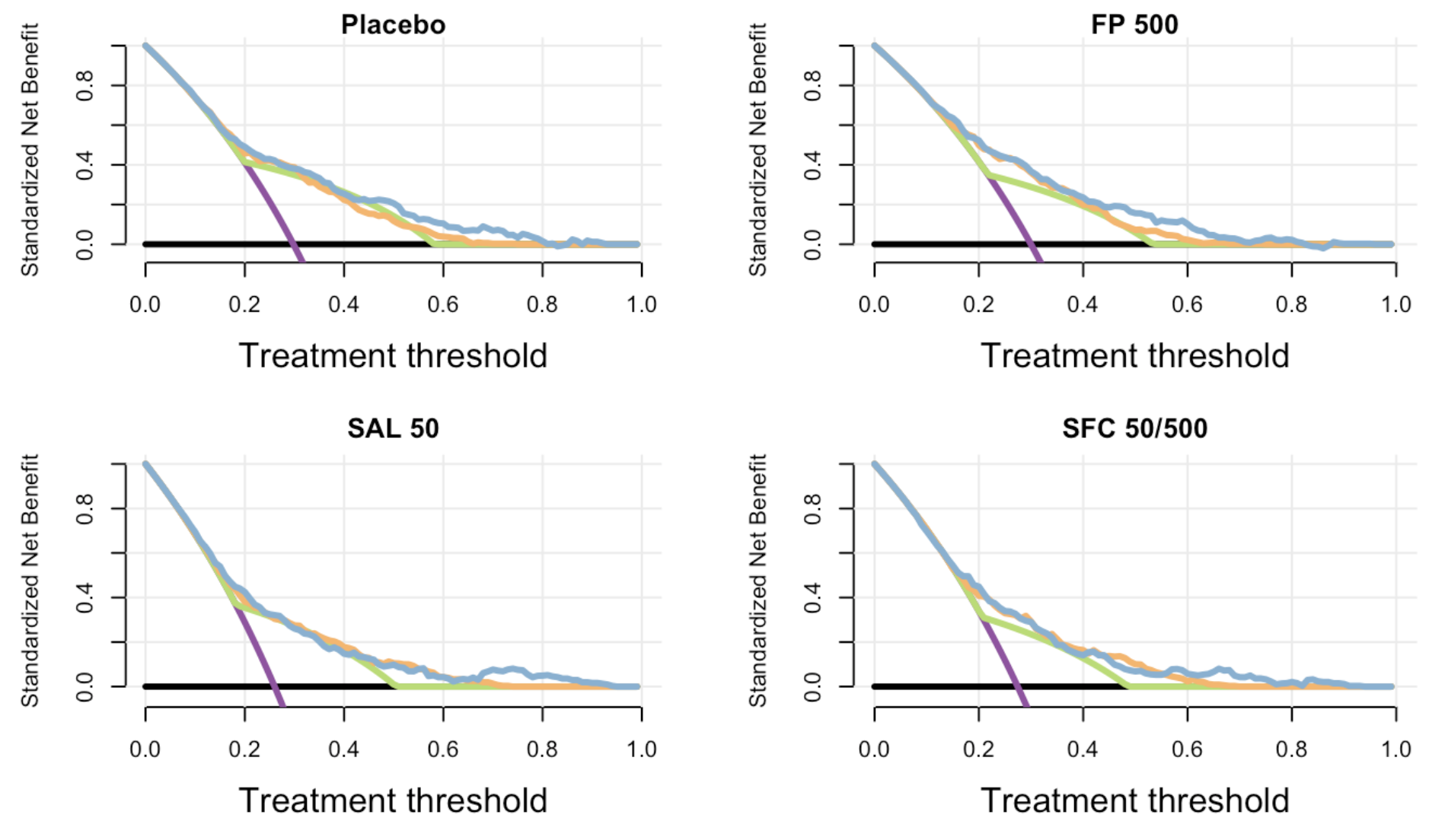


**Figure SM4**. Event: moderate/severe exacerbations; Patients: with history


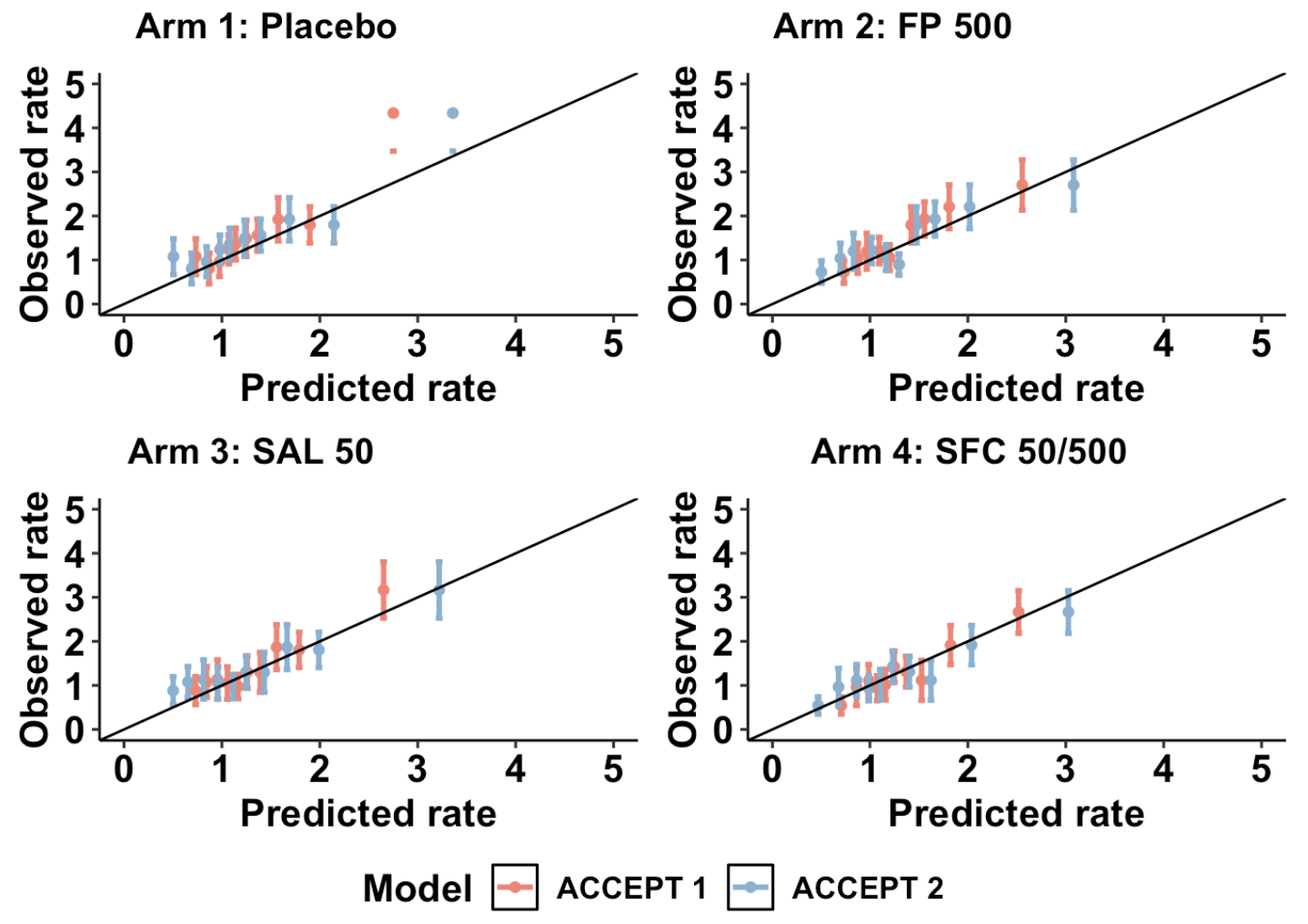


**Figure SM5**. Event: moderate/severe exacerbations; Patients: without history


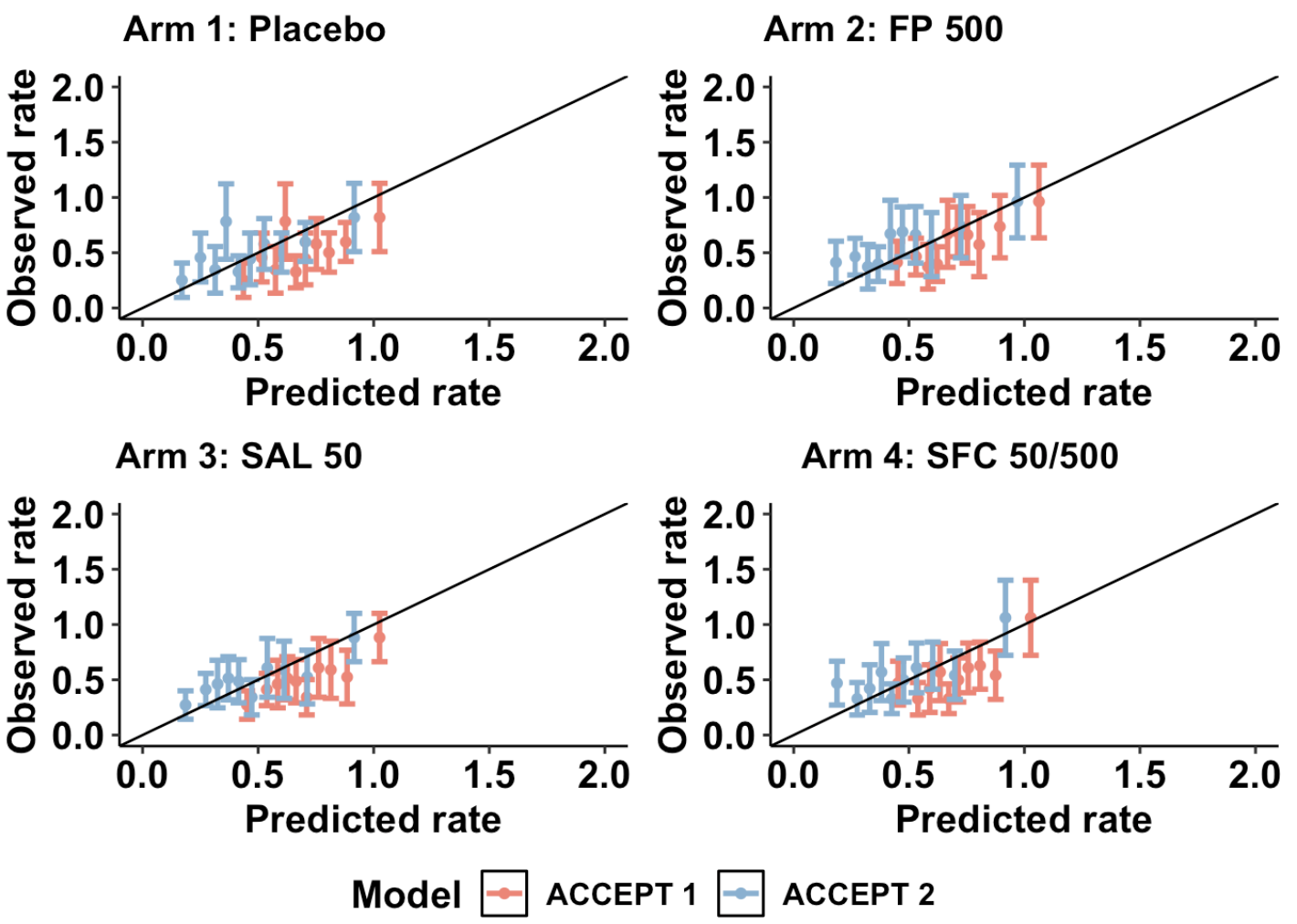


**Figure SM6**. Event: severe exacerbations; Patients: with history


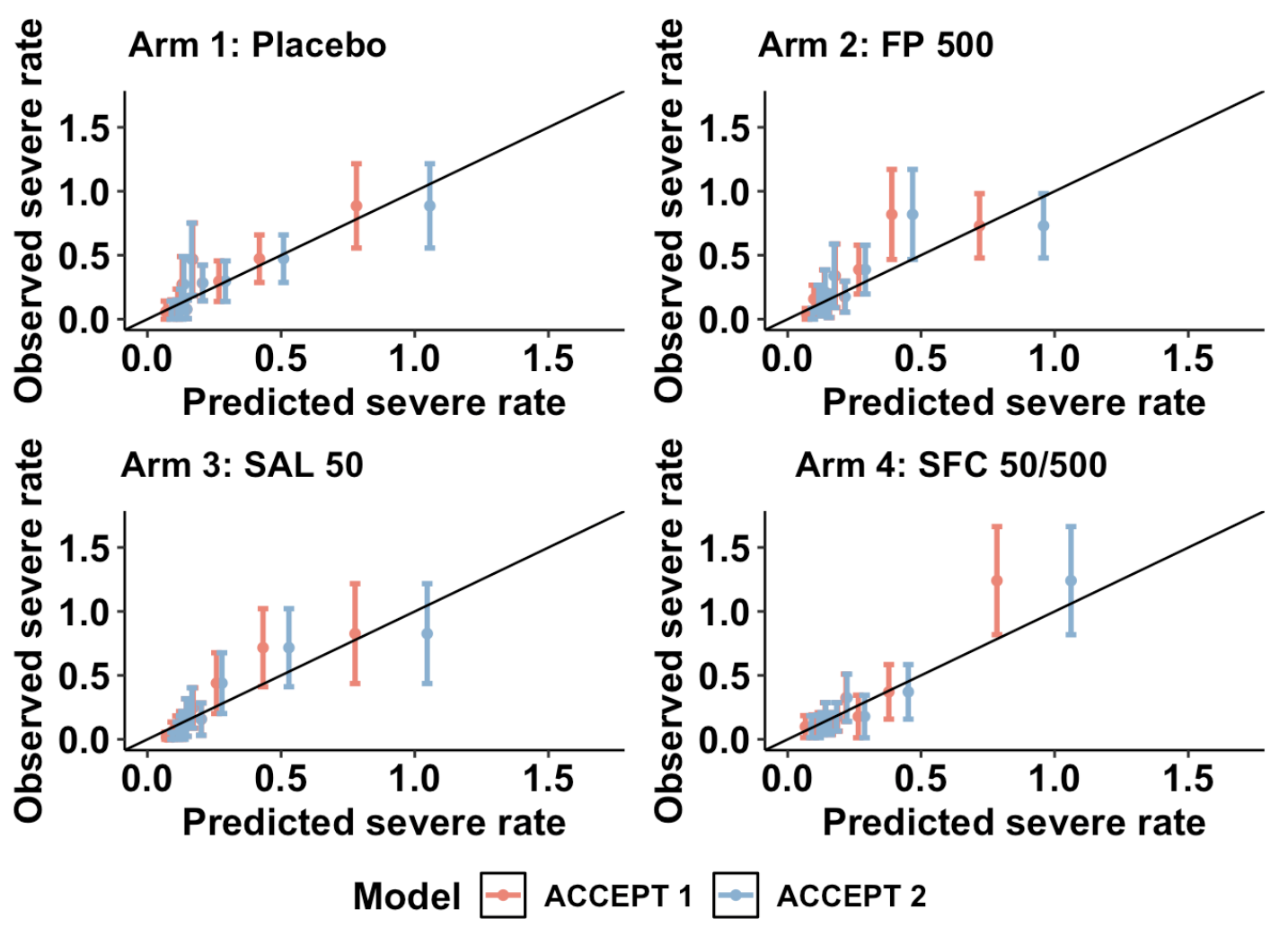


**Figure SM*7***. Event: severe exacerbations; Patients: without history


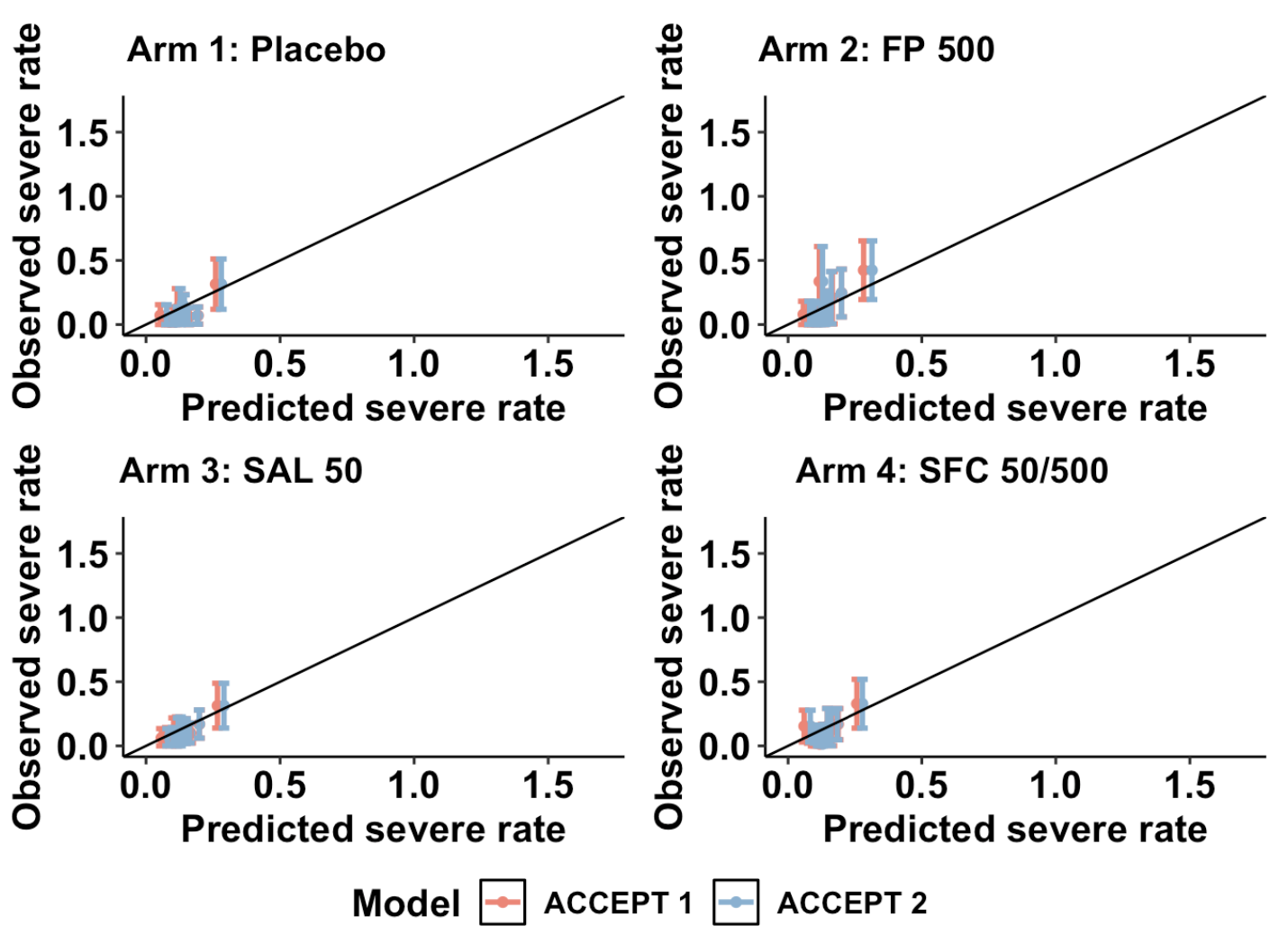


1. **Sensitivity analysis: no LAMA use in TORCH study**

**Figure SM*8***. Calibration plot of the predicted exacerbation rates in TORCH study.


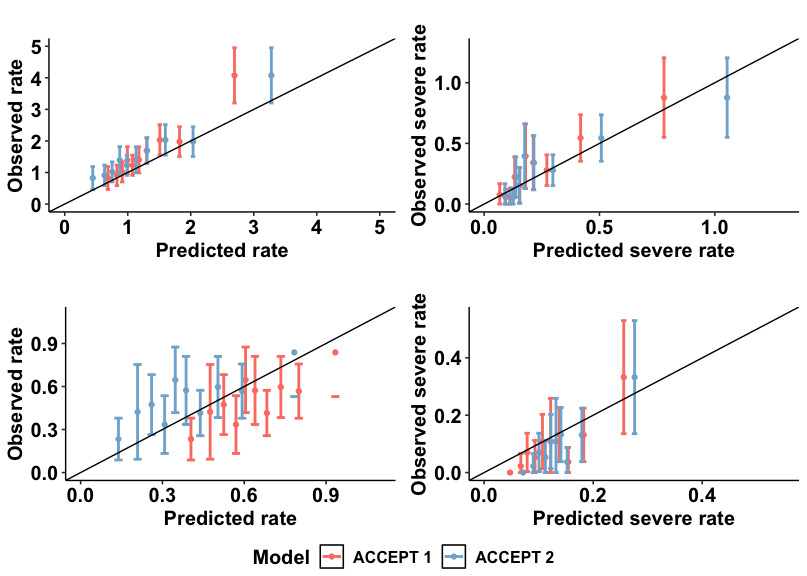


1. **R packages and functions**

Here is a list of R packages and functions used in our analyses:

- accept: fit ACCEPT 1 model
- splines: fit MARS model
- ggplot2 & ggpubr: calibration plots
- gmish: ICI analysis
- pROC and predtools: ROC curves and AUC
- timeROC: time dependent ROC curve and AUC
- rmda: DCA
