# Supplementary material for: ACCEPT 2·0: Recalibrating and externally validating the Acute COPD exacerbation prediction tool (ACCEPT)
Source: eClinicalMedicine. 2022 Jul 22;51:101574. doi: 10.1016/j.eclinm.2022.101574 (PMC9309408; doi:10.1016/j.eclinm.2022.101574)
Supplement: Supplementary file 2 [file mmc2.docx]

Appendix 1. IMPACT Study Group

| **Surname** | **First Name(s)** | **Affiliation** |
| --- | --- | --- |
| Bansback | Nick | School of Population and Public Health, University of British Columbia, Vancouver, British Columbia, Canada |
| Barn | Prabjit | Legacy for Airway Health, Vancouver Coastal Health Research Institute, Vancouver, British Columbia, Canada |
| Bottorff | Joan L. | 1. School of Nursing, University of British Columbia, Okanagan, British Columbia, Canada  2. Institute for Healthy Living and Chronic Disease Prevention, University of British Columbia, Okanagan, British Columbia, Canada |
| Bryan | Stirling | 1. Michael Smith Health Research BC, Vancouver, British Columbia, Canada  2. School of Population and Public Health, University of British Columbia, Vancouver, British Columbia, Canada |
| Burns | Paloma | Centre for Heart Lung Innovation, St. Paul’s Hospital and Department of Medicine (Division of Respirology), The University of British Columbia, Vancouver, Canada |
| Carlsten | Chris | 1. Centre for Lung Health, Vancouver Coastal Health Research Institute, Vancouver, British Columbia, Canada  2. Respiratory Medicine Division, Faculty of Medicine, University of British Columbia, Vancouver, British Columbia, Canada |
| Conklin | Annalijn I. | 1. Collaboration for Outcomes Research and Evaluation (CORE), Faculty of Pharmaceutical Sciences, University of British Columbia, Vancouver, British Columbia, Canada  2. Centre for Health Evaluation and Outcome Sciences (CHÉOS), St. Paul’s Hospital, Vancouver, British Columbia, Canada |
| De Vera | Mary | 1. Collaboration for Outcomes Research and Evaluation (CORE), Faculty of Pharmaceutical Sciences, University of British Columbia, Vancouver, British Columbia, Canada |
| Gershon | Andrea | Sunnybrook Research Institute, Toronto, Ontario, Canada |
| Gupta | Samir | 1. Li Ka Shing Knowledge Institute, St. Michael’s Hospital, Toronto, Ontario, Canada  2. Department of Medicine, University of Toronto, Toronto, Ontario, Canada |
| Gustafson | Paul | Department of Statistics, Faculty of Science, University of British Columbia, Vancouver, British Columbia, Canada |
| Hoens | Alison M. | Department of Physical Therapy, University of British Columbia, Vancouver, British Columbia, Canada |
| Mokhtaran | Mehrshad | Respiratory Evaluation Sciences Program, Collaboration for Outcomes Research and Evaluation, Faculty of Pharmaceutical Sciences, University of British Columbia, Vancouver, British Columbia, Canada |
| Johnson | Jim | Patient Partner |
| Joshi | Phalgun | Legacy for Airway Health, Vancouver Coastal Health Research Institute, Vancouver, British Columbia, Canada |
| Leung | Janice | Centre for Heart Lung Innovation, St. Paul’s Hospital and Department of Medicine (Division of Respirology), Faculty of Medicine, University of British Columbia, Vancouver, British Columbia, Canada |
| Lynd | Larry D. | 1.Collaboration for Outcomes Research and Evaluation (CORE), Faculty of Pharmaceutical Sciences, University of British Columbia, Vancouver, British Columbia, Canada  2. Centre for Health Evaluation and Outcome Sciences (CHÉOS), St. Paul’s Hospital, Vancouver, British Columbia, Canada |
| Metcalfe | Rebecca K. | 1.Centre for Health Evaluation and Outcome Sciences (CHÉOS), St. Paul’s Hospital, Vancouver, British Columbia, Canada  2. Respiratory Evaluation Sciences Program, Collaboration for Outcomes Research and Evaluation, Faculty of Pharmaceutical Sciences, University of British Columbia, Vancouver, British Columbia, Canada |
| Michaux | Kristina D. | Respiratory Evaluation Sciences Program, Collaboration for Outcomes Research and Evaluation, Faculty of Pharmaceutical Sciences, University of British Columbia, Vancouver, British Columbia, Canada |
| Sadatsafavi | Mohsen | 1. Respiratory Evaluation Sciences Program, Collaboration for Outcomes Research and Evaluation, Faculty of Pharmaceutical Sciences, University of British Columbia, Vancouver, British Columbia, Canada  2. Centre for Clinical Epidemiology and Evaluation, University of British Columbia, Vancouver, British Columbia, Canada |
| Simmers | Brian | Providence Health Care Authority, Vancouver, British Columbia, Canada |
| Sin | Don D. | Centre for Heart Lung Innovation, St. Paul’s Hospital and Department of Medicine (Division of Respirology), Faculty of Medicine, University of British Columbia, Vancouver, British Columbia, Canada |
| Smith | Daniel | Patient Partner |
| Struik | Laura | School of Nursing, University of British Columbia, Okanagan, British Columbia, Canada |
| Sutherland | Janet | Canadian Thoracic Society, Ottawa, Ontario, Canada |
| Vinay | Dhingra | 1. Vancouver Coastal Health Research Institute, Vancouver, BC, Canada  2. Department of Medicine, Faculty of Medicine, University of British Columbia, Vancouver, British Columbia, Canada |
